# A non-swellable, anisotropic hydrogel patch with superior mechanical stability for internal anti-adhesion via physical barrier and inflammation regulation

**DOI:** 10.1016/j.mtbio.2025.102017

**Published:** 2025-06-25

**Authors:** Jianyu Qiu, Xitong Kang, Tao Liu, Jing Liu, Huansheng Liu, Xiyang Zhao, Yong Li, Qi Liu, Zhenzhen Nong, Qingwen Wang, Zhenzhen Liu

**Affiliations:** a The Third Affiliated Hospital, School of Biomedical Engineering, Guangzhou Medical University, Guangzhou, 511436, China; b College of Food Science, South China Agricultural University, Guangzhou, 510642, China; c Institute of Biomass Engineering, College of Materials and Energy, South China Agricultural University, Guangzhou, 510642, China

**Keywords:** Anti-adhesion hydrogel, Anti-swelling, Post-operative adhesion, Tissue pro-healing, Inflammatory regulation

## Abstract

Tension-free repair operation in clinic needs an implantable mesh to repair the internal soft-tissue defect. However, the clinical patches fail to simultaneously achieve anti-adhesion, anti-deformation and tissue pro-healing. Herein, a high-performance anti-adhesion hydrogel patch is synthesized by one-step of directed freeze-casting technique via utilizing the FDA-approved poly (vinyl alcohol) (PVA) and carboxymethylcellulose nanofibril (CNF) as the network backbone without any chemical crosslinker. This fully physical crosslinked hydrogel exhibits remarkable mechanical performance because of the highly oriented microstructure and multiple hydrogen bonding, including the matching mechanical property with soft tissue, anti-tearing capability, the great 99 % retention of tensile stress and higher toughness after soaking at PBS for 7 days. And the extremely low swelling ratio of 5.9 % in PBS and combining the higher water-content is superior to other reported anti-swelling hydrogel. Importantly, besides as a robust physical barrier of this hydrogel patch, it also displays the excellent inflammatory regulation via capturing the positively charged proinflammatory cytokines because of the incorporation of the negatively charged CNF, synergistically promoting the remarkable anti-adhesion and tissue pro-healing efficiency. By integrating the anti-adhesion, tissue pro-healing, long-lasting anti-deformation in vivo and excellent bio-compatibility, this hydrogel provides a great candidate of anti-adhesion patch for tension-free repair operations.

## Highlights

• An anti-adhesive hydrogel was fabricated using one-step directional freeze-casting.
• Synergistic oriented structure/multi-hydrogen bonding enables 5.9 % swelling ratio.
• 99 % mechanical property was retained in hydrated environments.
• This hydrogel simultaneously achieves anti-adhesion and tissue regeneration.

## Graphical abstract

**Figure:**
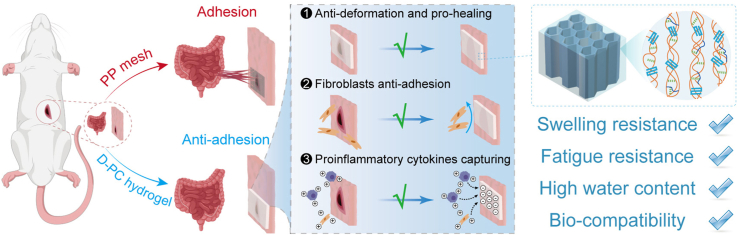

## 1 Introduction

Internal soft tissue injury inevitably occurs in clinic, because of oncologic resection, surgical trauma, soft tissue infection and so on. Especially, repairing internal defects of muscular layers and serosal membranes have attracted remarkable attentions in recent years, such as hernia repair. The tension-free repair operation which implanting the synthetic patches to the defect has been recommended as the popular approach, and displaying the significant advantages to the traditional surgical sutures [[1], [2], [3]]. However, the commercial patches, such as polypropylene (PP) and expanded polytetrafluoroethylene (e-PTFE), usually generate visceral adhesion which is easy to induce the severe complications of bowel obstruction, chronic pain, infertility, or even the failure of surgical hernia repair, seriously threatening the patient's life and health [4,5].

Developing superior anti-adhesion patches is the effective method to address the visceral adhesion issue [6]. Hydrogel is a 3D crosslinked soft material, which has high water-content, variable physio/chemical properties, similar tissue structure and biocompatibility, showing great advantages in tissue engineering field and has been extensively investigated [[7], [8], [9], [10]]. However, the high water-content of hydrogel commonly has poor mechanical strength and weak resilience which is difficult to withstand the internal abdominal wall pressure and easy to induce deformation [11]. Different strategies have been developed to reinforce the hydrogel, such as double-network [[12], [13], [14]], hydrophobic aggregation [15], incorporation of nanofiller [16,17], and phase separation [[18], [19], [20]]. The inspiring progress of tough hydrogels have been achieved, while as the internal anti-adhesion patch, except the high mechanical requirement, the non-swelling or low-swelling performance was also needed because of the constantly wet internal environment, especially in abdominopelvic cavity. However, most of the hydrogels display serious swelling in wet environment, which accompanied the large polymer expansion, deteriorated mechanical strength, subsequently separating from the defect and losing the barrier function [21,22]. Increasing crosslinking density [23], introducing hydrophobic monomer [24] or solvent exchange to rearrange the network structure [25], could improve the anti-swelling property of hydrogels, while the greatly increased polymer solid-content and non-aqueous solvent may have harmful effect to the biocompatibility which was another required key performance for the internal implanting materials.

Most biological tissues in living organisms have specific anisotropic structure. Human muscle is a typical example which possesses highly ordered orientation structure, endowing the muscle with great mechanical strength and excellent contraction capability [26]. Therefore, introducing highly ordered structure into the hydrogel patch would be effective for its robust mechanical performance yet keeping high water-content. Although a few anisotropic hydrogels have been reported recently via freeze-casting assisted salt out [27], freeze-casting followed by thermal annealing [28], bidirectional freeze-casting and subsequent compression annealing [29], or introducing crosslinked polymer into the wood skeleton [30], the tedious multi-step preparation or the incorporation of high-concentrated salt may limit their application in internal anti-adhesion patches. And the most reported anisotropic hydrogels were just focused on the improvement of their mechanical strength or toughness, the application of post-operative anti-adhesion has not been received attention. Moreover, besides the inherent bulk performance of patch, inflammation is a vital factor to promote the formation of visceral adhesion. Surgical trauma to the peritoneal epithelium could trigger a strong inflammatory response, involving the increasement of positively charged proinflammatory cytokines, such as IL-1β, IL-6, and TNF-α [31,32]. If the implanted anti-adhesion patch at injury site could effectively capture the excess positively charged pro-inflammatory cytokines to regulate the local inflammation, the visceral adhesion formation could be minimized or avoided. However, little attention has been given to the inflammation regulation function for the anti-adhesion patch, most are solely focused on the physical barrier role. Hence, developing a high-performance hydrogel patch integrated with high mechanical strength, non-swelling and mechanical stability in wet environment, excellent biocompatibility, and inflammation regulation capability is urgently needed.

**Fig. 1:**
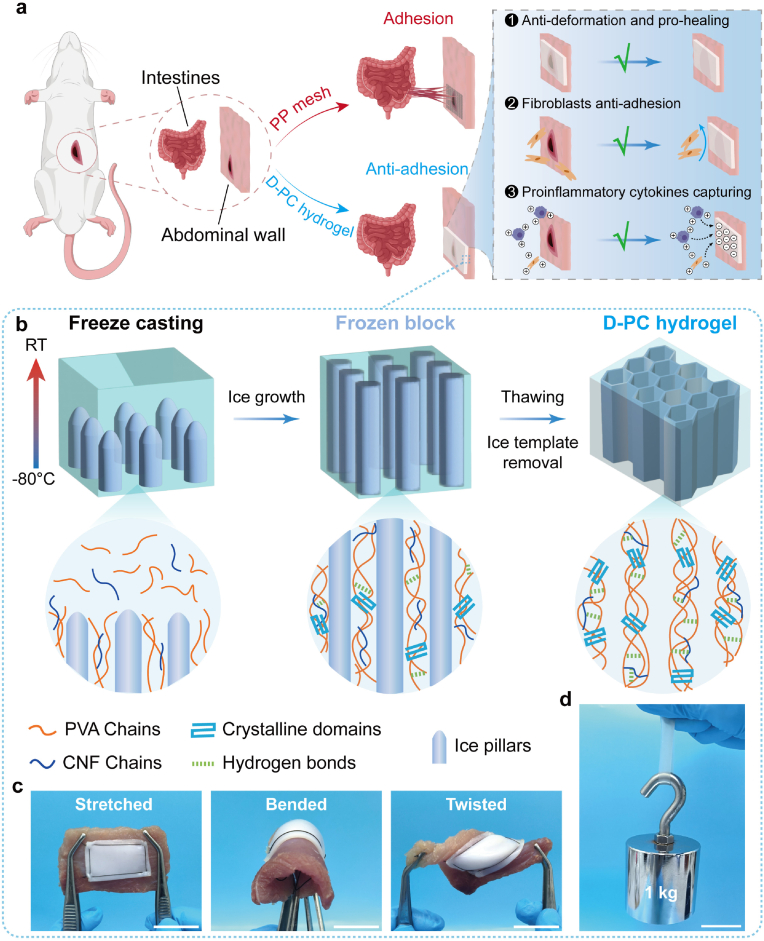
a) Schematic illustration of the effect of anti-adhesion for the D-PC hydrogel patch and the commercial PP mesh. b) Scheme for the fabrication of the hydrogel patch. c) Digital photos of the flexibility for D-PC hydrogel which was sutured on the porcine muscle. Scale bar: 2 cm. d) The image showing the high mechanical strength of the D-PC hydrogel along the freezing direction, which bearing a 1 kg weight. Scale bar: 2 cm.

In this work, a versatile hydrogel patch was designed to effectively prevent the visceral adhesion. A macromolecule of PVA and natural biomaterial of CNF which were approved by FDA were chosen as the hydrogel backbone and nanofiller, respectively. And the hydrogel patch was simply fabricated by freeze-casting, yet without any crosslinker or solvent immersion. On one hand, the synergistic effect of freeze-casting technique and the incorporation of CNF evidently improve the mechanical strength, toughness and swelling resistance of the resultant hydrogel. On the other hand, the negative charge of CNF endows the hydrogel patch with potential inflammation regulation capability which could be as a trap for positively charged pro-inflammatory cytokines (Fig. 1a). Interestingly, this hydrogel patch displays higher mechanical toughness after immersing in phosphate buffer saline (PBS, pH = 7.4) for one week, while simultaneously keeping the superior fatigue resistance, which was difficult to achieve for traditional hydrogels. Additionally, the extremely low swelling ratio of 5.9 % was superior to other reported anti-swelling hydrogel, simultaneously keeping high water-content of 89.8 %. Furthermore, the biocompatibility of hydrogel patch was systematically investigated, including the cell viability and hemolytic test. The anti-adhesion evaluation of hydrogel patch was conducted via cell adhesive test and establishing rat abdominal wall defect model by comparing with the commercial anti-adhesion patch. The results demonstrate this hydrogel patch could effectively prevent the visceral adhesion in vivo not only playing the role of physical barrier because of the robust mechanical stability, but also regulating the inflammatory microenvironment by capturing the positively charged pro-inflammatory cytokines. This work provides a promising anti-adhesion patch candidate for clinical tension-free soft-tissue repair.

## 2 Experimental section

### 2.1 Materials

PVA powder (M_W_ = 130,000 Da, 90,000 Da, 160,000 Da, 99 % hydrolyzed, Sigma-Aldrich) and carboxymethylcellulose nanofiber (CNF, 1 % aqueous dispersion, Guilin Qihong Science and Technology Co.) were used. The acrylic molds for the experiments were homemade. Deionized water was used throughout the experiment.

### 2.2 Preparation of D-PC hydrogel

The D-P_10_C_2_ hydrogel was prepared as follows. PVA powder (2g) and CNF solution (4g) were dissolved in deionized water (14 mL) at 95 °C. The obtained solution was poured into an acrylic mold after degassing. To ensure the vertical growth of ice crystals and form parallel aligned ice crystals, the molds were positioned horizontally on the surface of a cold copper plate. The copper plate was cooled by liquid nitrogen at a rate of 2 °C min^−1^ to −80 °C. After the freezing process, the samples were transferred to a refrigerator at −20 °C for 24 h and thawed at room temperature (25 °C). The obtained hydrogels were D- P_10_C_2_ hydrogels.

### 2.3 Preparation of R- P_10_C_2_ hydrogel

PVA powder (2g) and CNF solution (4g) were added to deionized water (14 mL) and dissolved at 95 °C. The obtained solution was poured into an acrylic mold after degassing. The molds were placed in a −20 °C refrigerator until the freezing process was completed and thawed at room temperature (25 °C). The obtained hydrogels were R-P_10_C_2_ hydrogels.

### 2.4 SEM characterization

Internal microstructure of the hydrogels was observed via a scanning electron microscope (EVO MA 15, Zeiss). All hydrogel samples were freeze-dried after being immersed in liquid nitrogen and fractured either perpendicular or parallel to the direction of ice crystal growth. The fractured surfaces were coated with gold and analyzed by scanning electron microscopy.

### 2.5 DIC measurement

Digital Image Correlation (DIC) technique was used to monitor the tensile test of hydrogels, a high-speed video camera (DH1200, Revealer) was used to record the whole process. First, spots were created by spraying paint on the hydrogel surface, followed by stretching of the hydrogel samples at a rate of 10 mm min^−1^. The DIC images were analyzed via RDIC software.

### 2.6 SAXS measurement

The microstructural differences of D-P_10_C_2_ and R-P_10_C_2_ hydrogels were observed via small angle X-ray scattering (SAXS) tests, the hydrogels were stretched as needed. The experiments were performed using an X-ray small angle scattering device (Xeuss 2.0, xenocs). The light source of the equipment is a copper-targeted X-ray tube running at 30 W with an X-ray wavelength of 1.54189 Å.

### 2.7 Zeta potential measurements

The freeze-dried hydrogel samples (*n* = 4) were ground into small particles. 5 mg of particles were weighed and diluted with 1 mL of deionized water. The zeta potential of the hydrogel solution was determined using a zinc meter (Zatasizer Nano ZS 90, Malvern).

### 2.8 Swelling ratio test

To evaluate the swelling ratio of the hydrogel, a hydrogel with a dimension of 9 mm (L), 9 mm (W) and 1.5 mm (t) was immersed in PBS solution (pH = 7.4) at 37 °C. The samples were removed at different intervals and their weights (noted as W″) were recorded. The swelling ratio (SR%) was calculated according to the following formula:

(1) $$\text{Swelling}\;\text{ratio}\;\left(\%\right)=\left(W^{\prime \prime }-W^{\prime }\right)/W^{\prime }\times 100\%$$

where W′ and W″ represent the initial weight and weight at different time intervals of the hydrogel samples, respectively.

### 2.9 Tensile test

All tensile tests were performed using an electronic universal testing machine (CMT-1203, Sansitaijie, maximum load 2 kN) at a constant rate of 30 mm min^−1^. For D-PC ∥ samples, the microstructure was parallel to the tensile force direction, and for D-PC ⊥ samples, the microstructure was perpendicular to the force direction. Hydrogels were cut into dumbbell shapes with dimensions of 35 mm (L), 2 mm (W) and 2 mm (t). For solubilized hydrogels, the samples were immersed in PBS (pH = 7.4) at 37 °C for 7 days and then stretched at a rate of 30 mm min^−1^. In continuous loading and unloading tensile tests, the samples were loaded and unloaded continuously at different strains (50 %–300 %). Cyclic tensile tests fixing 50 % strain were performed for 100 cycles at a tensile rate of 10 mm min^−1^.

### 2.10 Pure shear test

Pure shear tests were performed using an electronic universal testing machine (CMT-1203, Sansitaijie, maximum load 2 kN) to quantitatively measure the shear stress. The test specimens were rectangular in shape with dimensions of 40 mm (L), 20 mm (W) and 2 mm (t) and were categorized into notched and unnotched types. The notch was in the middle of the specimen's edge (5 mm in length), which was cut using a single-edged blade (Gillette, Shanghai). Shear stress-strain curves were obtained by stretching the sample at 10 mm min^−1^.

### 2.11 Suture tensile test

In order to simulate the suture conditions after hernia patch implantation and to facilitate mechanical testing, a simplified continuous suture technique was used. D-P_10_C_2_ and R-P_10_C_2_ hydrogels were each secured between two tensile fixtures using 4-0 surgical sutures. According to the standard procedures for surgical patch implantation, the distance between the suture loops was set at 1 cm, and a gap of 0.5 cm was maintained from the edge of the sample to the suture loops.

### 2.12 Cell adhesion evaluation

A live/dead staining assay was performed using L929 fibroblasts on a hydrogel in a solubilized equilibrium state to evaluate cell adhesion capacity. The hydrogels were positioned in a 24-well plate and immersed with MEM medium (supplemented with 10 % fetal bovine serum and 1 % penicillin-streptomycin) for 1 h. L929 cells were seeded on the hydrogel surfaces at a density of 8 × 10^4^ cells per well and cultured in a 37 °C, 5 % CO_2_ incubator for 24 h. The medium was removed and the samples were carefully washed three times with PBS. The samples were then stained with calcein-AM and the stained cells were observed via a fluorescence microscope (Axio Observer A1, Zeiss). MEM medium (supplemented with 10 % fetal bovine serum and 1 % penicillin-streptomycin) was served as a control.

### 2.13 Hemolysis assay

The hemolysis rate was assessed by adding hydrogel extract (500 μL) to 500 μL of 4 % rabbit erythrocyte suspension. PBS was served as a negative control and deionized water as a positive control. After incubation at 37 °C for 2 h, the suspension was collected and centrifuged (2000 rpm, 2 min). 100 μL of supernatant from each group was transferred to a 96-well plate separately and absorbance measurements were performed using an enzyme marker (540 nm). The hemolysis percentage was calculated using the following formula:

(2) $$\text{Hemolysis}\;\text{ratio}\;\left(\%\right)=\left(A_{h}-A_{n}\right)/\left(A_{p}-A_{n}\right)\times 100\%$$

where A_h_, A_p_ and A_n_ represent the absorbance values of D- P_10_C_2_ hydrogel group, positive control and negative control, respectively.

### 2.14 In vitro cytotoxicity test

L929 fibroblast was used to evaluate the cytocompatibility of D-P_10_C_2_ hydrogels. The extract was prepared by immersing 15 mg of dehydrated D-P_10_C_2_ hydrogel in 3 mL of MEM medium (supplemented with 10 % fetal bovine serum and 1 % penicillin-streptomycin). L929 cells were cultured in a 96-well plate at a density of 8 × 10^3^ cells per well and incubated at 37 °C, 5 % CO_2_ for 24 h. Subsequently, the medium was replaced with D- P_10_C_2_ hydrogel extract for an additional 24-h incubation at 37 °C, 5 % CO_2_. MEM medium (supplemented with 10 % fetal bovine serum and 1 % penicillin-streptomycin) was served as a control group. Five replicates were set up for each experimental group (*n* = 5). Cell viability was assessed via the CCK-8 assay. Live/dead cells were stained using calcein AM/PI staining kit (provided by Beyotime). The stained cells were observed and photographed using a fluorescence microscope (Axio Observer A1, Zeiss).

### 2.15 In vivo studies of the hydrogel patches

Animal experiments were conducted at the Laboratory Animal Center of South China Agricultural University and approved by the Laboratory Animal Ethics Committee (animal experimentation ethics number: 2024b059). SD rats weighing between 200 ± 20 g were selected for the establishment of the abdominal wall defect model. Rats were anesthetized with 3 % sodium pentobarbital. Subsequently, a circular defect measuring 8 mm in diameter was created in the abdominal wall under aseptic surgical technique with a standard biopsy punch. A D-P_10_C_2_ hydrogel patch and a commercial PP mesh (purchased from Changzhou Medical Equipment General Factory Co. Ltd) were sutured to cover the area of the injured abdominal wall with a 4-0 suture, respectively. Finally, the abdominal wall and skin were sutured sequentially through 4-0 sutures. The rats were housed in the controlled animal facilities with relative humidity at 55–60 % and temperature conditions at 22–25 °C under 12/12-h light/dark cycle, with access to food and water ad libitum. Each animal was used for only one experiment.

### 2.16 Morphological observation and pathological analysis

The rats were euthanized at the respective endpoint via intraperitoneal injection of an excessive amount of anesthetic. Subsequently, the skin was dissected and sutures were reopened to reveal the repair site to evaluate the adhesion formation and the defect healing progress. The repaired areas were photographed and preserved in formaldehyde, followed by HE staining, Masson trichrome staining, and immunohistochemical staining for CD68, IL-1β, and TNF-α. Histologic images were evaluated by a blinded pathologist and quantitatively analyzed using ImageJ software.

### 2.17 Statistical analysis

Data were presented as mean ± standard deviation (SD). Statistical analyses were performed through GraphPad Prism version 9.5. T-test was applied for statistical analysis. Statistical significance was expressed as ∗ p < 0.05, ∗∗p < 0.01 and ∗∗∗p < 0.001.

## 3 Results and discussion

### 3.1 Preparation and mechanical characterization of hydrogel patch

The freeze-casting strategy was illustrated in Fig. 1b. A mixed solution of PVA and CNF was casted into a home-made acrylic mold and directionally frozen on a cold substrate at −80 °C following a vertical temperature gradient under a certain cooling rate. The polymer phase of PVA and CNF was separated from the solution because of the unidirectional crystallization of ice pillars, facilitating the entanglement of PVA and CNF chains. Due to the abundant polar groups on the PVA (-OH groups) and CNF (-OH, -COOH groups) molecular chains, the multiple hydrogen-bonding were generated between the PVA and CNF chains, providing the initial crosslinking. Subsequently, this frozen block was put at −20 °C for 24 h to induce much more crystallization. Finally, the mold was removed and obtaining the hydrogel which was labeled as D-P_*x*_C_*y*_ hydrogel, *x* represents the weight content of PVA in the resultant hydrogel, and *y* represents the weight ratio of CNF to PVA. Meanwhile, the randomly structured hydrogel (R-P_*x*_C_*y*_) was prepared to be as the control sample which was directly froze at −20 °C without the gradient cooling process. As shown in Fig. 1c–d, this D-PC hydrogel was pliable and easy to be fixed at the tissue surface by suturing, and still kept excellent stability after different kinds of twisted stimulus and withstanding the 1 kg weight. These great mechanical performances are suitable for the flexible operation of clinical abdominal surgery.

**Fig. 2:**
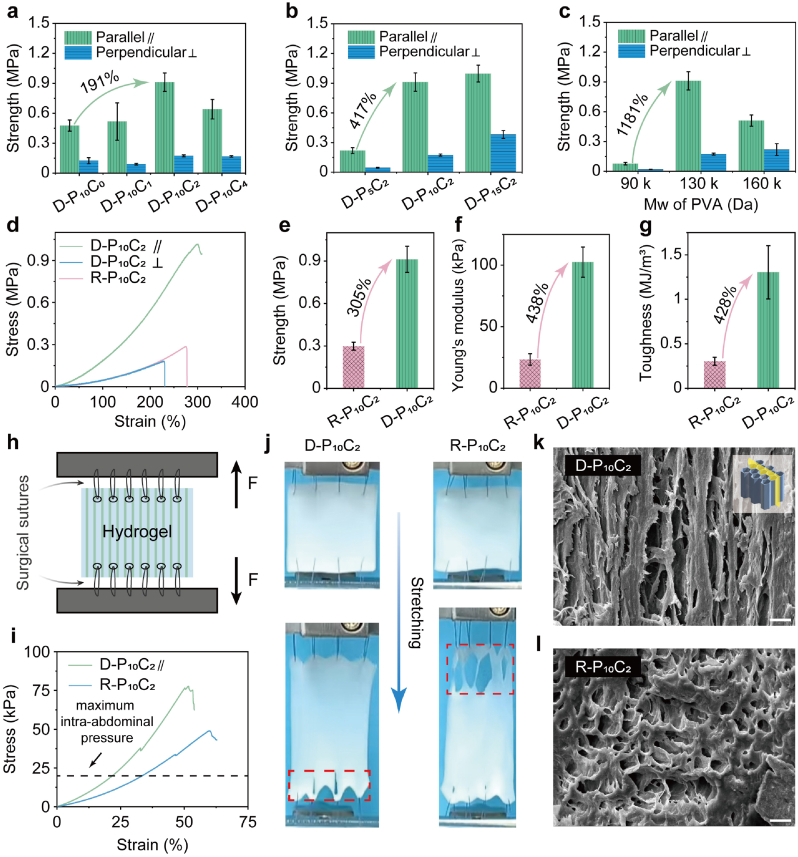
a) The tensile stress of the D-PC hydrogels with different contents of CNF (the hydrogels were stretched along the parallel and vertical directions to freezing, labeled as Parallel ∥ and Perpendicular ⊥, respectively). b) The tensile stress of the D-PC hydrogels with different contents of PVA. c) The tensile stress of the D-PC hydrogels with different molecular weight of PVA. d) Tensile stress-strain curves of D-P_10_C_2_ and R-P_10_C_2_ hydrogels. e) The tensile stress of the D-P_10_C_2_ and R-P_10_C_2_ hydrogels. f) Young's modulus of D-P_10_C_2_ and R-P_10_C_2_ hydrogels. g) The toughness of D-P_10_C_2_ and R-P_10_C_2_ hydrogels. h) Schematic illustration of the suture stretching test of the hydrogels. i) Suture tensile stress-strain curves of D-P_10_C_2_ and R-P_10_C_2_ hydrogels. j) Digital photos of the suture stretching of the D-P_10_C_2_ and R-P_10_C_2_ hydrogels. k) SEM images of the D-P_10_C_2_ hydrogels parallel to the freezing direction. Scale bar: 10 μm. l) SEM images of the R-P_10_C_2_ hydrogels. Scale bar: 10 μm.

The mechanical property of D-PC hydrogel was systematically evaluated by the tensile tests, including the perspectives perpendicular and parallel to the freezing direction. As shown in Fig. 2a–c, the tensile stress of parallel direction for different formulated D-PC hydrogels was much higher than that of perpendicular direction, displaying the evident anisotropic property. Notably, the additive content of CNF, the weight concentration and the molecular weight (M_W_) of PVA, both play key role for the mechanical performance of the resultant D-PC hydrogel. As increasing the content of CNF when fixing the content of PVA was 10 wt %, the tensile stress of D-PC hydrogel first increased and then decreased. The maximum stress was 0.91 MPa (D-P_10_C_2_) which displayed 191 % increasement compared to that of D-P_10_C_0_, demonstrating the much more hydrogen bonding interactions were generated between the CNF and PVA molecular chains. While the stress of D-P_10_C_4_ decreased, because the much more incorporation of CNF could disrupt the crystalline structure of PVA. Although the tensile stress increased as increasing the weight content of PVA, the optimized sample was D-P_10_C_2_ because of the difficult operation ascribed to the high viscosity of D-P_15_C_2_, displaying 417 % increasement compared to D-P_5_C_2_. The molecular weight of PVA was optimized as 130 kDa, which possessing 1181 % increasement of tensile stress compared to that of 90 kDa, ascribing to the appropriate structure that generating the most interactions for the hydrogel network. Notably, the mechanical performance of D-P_10_C_2_ was superior to that of random R-P_10_C_2_ (Fig. 2d), including the 305 % increasement of tensile stress (Figs. 2e), 438 % increasement of Young's modulus (Figs. 2f) and 428 % increasement of toughness (Fig. 2g). And the Young's modulus of this optimized D-P_10_C_2_ (102 kPa) was similar to that of abdominal wall (80 kPa) [32], this good mechanical match could effectively decrease the foreign body reaction (FBR) of the implants. As an abdominal anti-adhesion patch, it must resist the abdominal pressure at the sutured state [33]. Hence, a mimic experiment was conducted to detect the influence of surgical sutures on the tensile behavior of this D-P_10_C_2_ patch (Fig. 2h). As shown in Fig. 2i, the surgical sutures were separated from the D-P_10_C_2_ hydrogel until the tensile stress was 76.5 kPa, which was much higher than the maximum intra-abdominal pressure for ordinary individuals (20 kPa) [31]. And Fig. 2j showed just a little deformation was observed for D-P_10_C_2_ hydrogel when stretching the suture lines, while a severe destruction happened for R-P_10_C_2_ hydrogel, demonstrating the superior tear resistance of D-P_10_C_2_ hydrogel (Movie S1-2). The superior mechanical performances of D-P_10_C_2_ hydrogel compared to R-P_10_C_2_ hydrogel, especially the freezing direction, are attributed to its hierarchical structure, which featured an aggregated, anisotropic microstructure with crosslinked sub-micron fiber bundles. In contrast to the random 3D network of R-P_10_C_2_ hydrogel, this highly oriented microstructure of D-P_10_C_2_ hydrogel could be visually verified by scanning electron microscope (SEM) images (Fig. 2k–l).

**Fig. 3:**
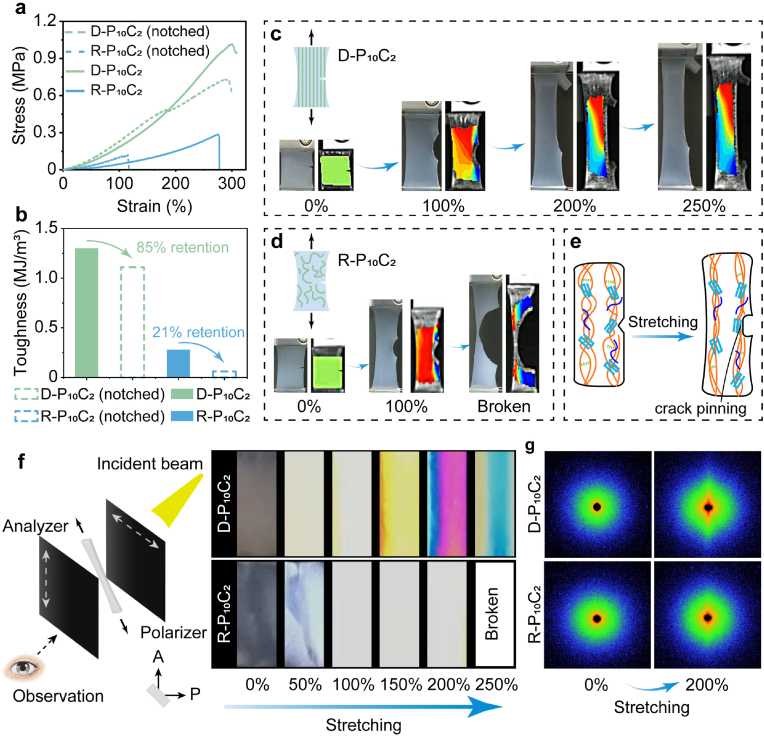
a) Tensile stress-strain curves of notched and unnotched D-P_10_C_2_ and R-P_10_C_2_ hydrogels. b) The toughness of notched and unnotched D-P_10_C_2_ and R-P_10_C_2_ hydrogels. The development of notch for D-P_10_C_2_ (c) and R-P_10_C_2_ (d) hydrogel and the corresponding DIC images. e) Schematic illustration of the tear resistance of D-P_10_C_2_ hydrogel. f) Schematic illustration of hydrogel stretching test between the crossed polarizers and the corresponding photos for D-P_10_C_2_ and R-P_10_C_2_ hydrogels under different tensile strain. g) SAXS patterns for the D-P_10_C_2_ and R-P_10_C_2_ hydrogels.

In order to further validate the significant enhancement along the freezing direction of D-P_10_C_2_ hydrogel, a notch (5 mm) was made and the fracture behavior was investigated by tensile stress-strain test. The samples of D-P_10_C_2_ and R-P_10_C_2_ hydrogels were precut perpendicular to the freezing direction. Fig. 3a shows the shearing curves of both notched and unnotched samples. Surprisingly, the notched D-P_10_C_2_ hydrogel demonstrated a remarkable crack-blunting ability, the toughness of notched sample was just a little bit lower than that of unnotched sample (Fig. 3b). The different state of notched hydrogel sample was observed as gradually increasing the stretching strain (Movie S3-4). It was found that the cracks for D-P_10_C_2_ hydrogel did not propagate along with the notch at the point of stress concentration until reaching the fracture strain which could also be clearly demonstrated by digital image correlation (DIC) (Fig. 3c–d). While the notch generated fatal destruction for R-P_10_C_2_ hydrogel, the toughness of notched sample was just 21 % of that of unnotched sample, and crack quickly propagated and completely destroyed the sample at a small strain of 180 %. This great crack-blunting capability was ascribed to the anisotropically aligned fiber bundle structures, where aligned fiber bundles delay the fracture through crack pinning (Fig. 3e). Additionally, the beautiful iridescent birefringence patterns under polarized light for the stretching D-P_10_C_2_ hydrogel also verified the anisotropic structure of this kind hydrogel (Fig. 3f). Meanwhile, small-angle X-ray scattering (SAXS) was also utilized to characterize the anisotropic structure of hydrogel which was stretched parallel to the freezing direction (Fig. 3g and S1). Fig. 3g showed a much sharper elongated equatorial streak appeared for D-P_10_C_2_ hydrogel at 200 % tensile strain, demonstrating the much easier orientation of molecular chains at tensile stress because of the oriented structure for D-P_10_C_2_ hydrogel.

### 3.2 Anti-swelling performance of this hydrogel patch

**Fig. 4:**
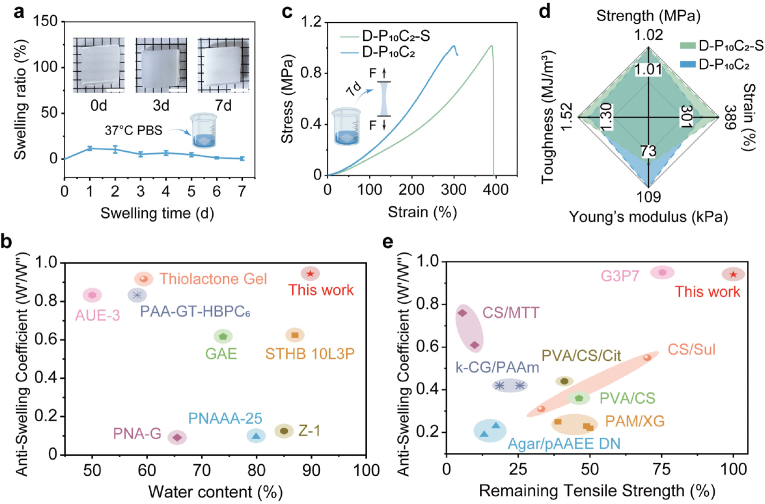
a) Swelling ratios of D-P_10_C_2_ hydrogel immersed in PBS buffer over 7 days and the corresponding photos of the swelled D-P_10_C_2_ hydrogel at different time intervals. Scale bar: 4 mm. b) Ashby chart of the anti-swelling coefficient versus water content among various hydrogels. c) Tensile stress-strain curves of D-P_10_C_2_ and D-P_10_C_2_-S hydrogels. d) Comparison in tensile stress, tensile strain, Young's modulus and toughness of the D-P_10_C_2_ and D-P_10_C_2_-S hydrogels. e) Ashby chart of the anti-swelling coefficient versus remaining tensile stress among various hydrogels.

Besides possessing the initially great mechanical strength, the anti-swelling ability is also vital for the durability of an anti-adhesion patch in vivo. Hence, the anti-swelling performance of D-P_10_C_2_ hydrogel was systematically investigated. As shown in Fig. 4a, when the D-P_10_C_2_ hydrogel was soaked into PBS solution (pH = 7.4, 37 °C), it reaches the highest swelling ratio of 11 % at first 1 day, then gradually decreases until equilibrium (5.9 %) within 7 days. This extremely low swelling ratio of 5.9 % but simultaneously keeping the high water-content (89.8 %) of the D-P_10_C_2_ hydrogel demonstrates its superior anti-swelling capability compared to other reported hydrogel barriers (Fig. 4b) [[34], [35], [36], [37], [38], [39], [40], [41]]. Surprisingly, the D-P_10_C_2_-S (the swollen D-P_10_C_2_ hydrogel after immersing in PBS for 7 d) hydrogel which was immersed at PBS (37 °C) for 7 days showed a little shrinkage of volume and became much opaquer compared to the unsoaked D-P_10_C_2_ hydrogel (the inset of Fig. 4a), and the swelling performance of D-P_10_C_2_ in di-H_2_O displayed the different behavior of evident volume expansion (Fig. S2), demonstrating the Hofmeister effect which the ions (K^+^, Na^+^, Cl^−^) in PBS buffer could induce the aggregation of PVA chains and reinforce the hydrogel network. Meanwhile, the swelling ratio of R-P_10_C_2_ hydrogel in 37 °C PBS was higher than that of D-P_10_C_2_ hydrogel in the same condition (Fig. S3), ascribing to the effect of the oriented microstructure for D-P_10_C_2_ hydrogel. The comprehensive mechanical performance of D-P_10_C_2_ hydrogel before and after incubation was quantitatively compared (Fig. 4c–d). The D-P_10_C_2_-S exhibits longer tensile strain, higher toughness, and comparable tensile stress and Young's modulus, demonstrating the remarkable anti-swelling and anti-deformation ability of D-P_10_C_2_ hydrogel. Compared to many reported hydrogel materials, this work exhibited impressive anti-swelling capability and mechanical strength stability [32,[42], [43], [44], [45], [46], [47], [48]], which is suitable for long-lasting physical barrier in vivo (Fig. 4e).

**Fig. 5:**
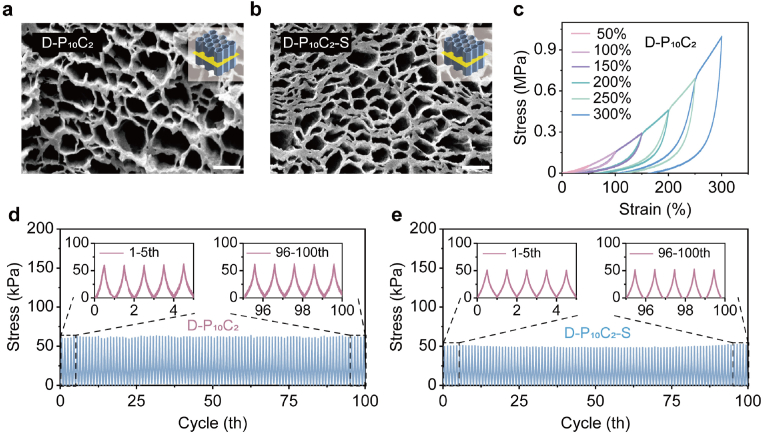
SEM images of pore size for the D-P_10_C_2_ (a) and the D-P_10_C_2_-S hydrogels (b), Scale bar: 5 μm. c) Cyclic tensile stress-strain curves of D-P_10_C_2_ hydrogel corresponding to different strains. Cyclic tensile loading-unloading curves of the D-P_10_C_2_ (d) and D-P_10_C_2_-S (e) hydrogel at 50 % strain.

Furthermore, the microstructure of D-P_10_C_2_ hydrogel and D-P_10_C_2_-S hydrogel were detected by SEM, respectively. The honeycomb microstructure of Fig. 5a clearly demonstrates the loosely porous structure, and the smaller pores of D-P_10_C_2_-S hydrogel sample verifies excellent anti-swelling performance of D-P_10_C_2_ hydrogel (Fig. 5b). Furthermore, owning good anti-fatigue property for the hernia patch is beneficial for resisting the dynamic abnormal pressure. The loading-unloading tensile tests were conducted to evaluate the anti-fatigue performance of D-P_10_C_2_ hydrogel. The tensile stress stepwise increases as increasing the tensile strain from 50 % to 300 %, and the hysteresis loop is barely evident at small strains (Fig. 5c). Additionally, the tensile stress could keep constant when the D-P_10_C_2_ hydrogel was successively stretched at least 100th cycles, demonstrating the better energy dissipation during the repeated loading-unloading testes because of the highly orientated microstructure and abundant hydrogen bonding interactions (Fig. 5d). Importantly, the soaked hydrogel of D-P_10_C_2_-S also showed the excellent tensile stability after 100th stretching test, and the stress remained kept at 50 kPa, because of the Hofmeister effect of PVA in PBS buffer solution which induced much more aggregation and hydrogen bonding interactions of molecular chains (Fig. 5e). And the stress of 50 kPa is much higher than that of abdominal pressure for an individual even at sneeze or cough state (20 kPa) [31], further verifying the remarkable anti-swelling performance and the anti-fatigue capability of this D-P_10_C_2_ hydrogel, and strongly demonstrating its potential to resist the internal wet environment and continuously dynamic abdominal pressure as a hernia patch.

### 3.3 In vitro biocompatibility and cell adhesion characterization of the hydrogel patch

**Fig. 6:**
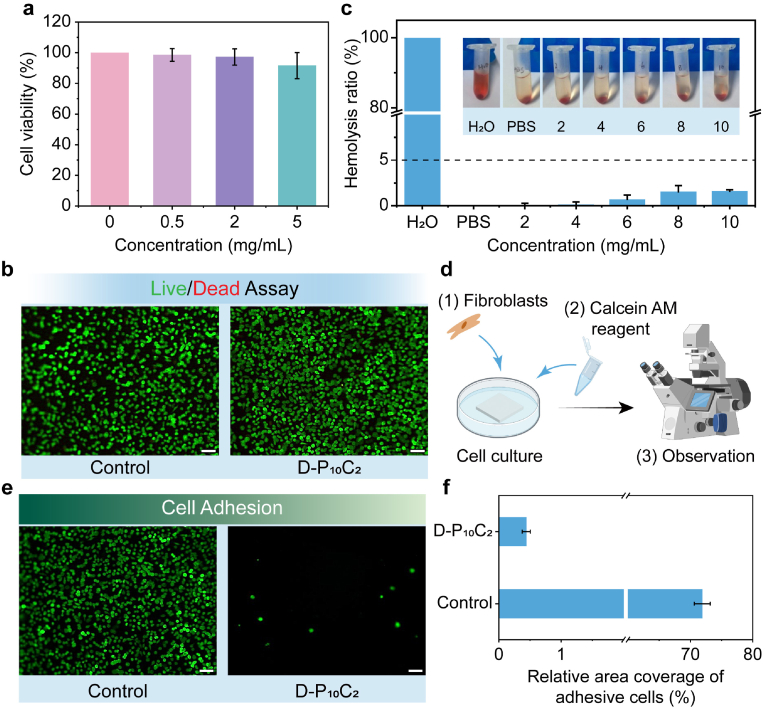
a) L929 cells viabilities treated with D-P_10_C_2_ hydrogel with different concentrations. b) Live/dead staining images of L929 cells after contacting with D-P_10_C_2_ hydrogel. Scale bar: 200 μm. c) Hemolysis ratios and photos of the blood compatibility of D-P_10_C_2_ hydrogels. d) Schematic illustration of the cell adhesion test for the hydrogels. e) Fluorescence images of L929 cells cultured on MEM complete medium and D-P_10_C_2_ hydrogel. Scale bar: 100 μm. f) The relative area of L929 cells cultured on D-P_10_C_2_ hydrogel and MEM complete medium.

Excellent biocompatibility is the precondition of a bio-medical material. The cellular cytocompatibility of the D-P_10_C_2_ hydrogel was firstly assessed by CCK8 and Live/Dead staining by coculturing L929 cell for 24 h. As shown in Fig. 6a, the cell viability of hydrogel sample was comparable to that of control sample when the concentration of hydrogel sample was 0.5 and 2 mg mL^−1^. Even for the high concentration of 5 mg mL^−1^, it still did not display cytotoxicity. And the Live/Dead staining assay shows the comparable cellular morphology and density, and no evident red staining dead cells, also demonstrating the excellent cytocompatibility of this D-P_10_C_2_ hydrogel (Fig. 6b). Furthermore, the hemocompatibility was conducted to verify the hemocompatibility of hydrogel sample. Fig. 6c showed the severe hemolysis generated for the positive control (H_2_O) which was characterized by a dramatic color change from colorless to the bright red. While for the negative control (PBS) and hydrogel treated groups (from 2 to 10 mg mL^−1^), just pale yellow and transparent supernatant were tested and the quantitatively calculated hemolysis ratios were below 2 %, which were in permissible international standard limit range. The above great cytocompatibility and hemocompatiblity demonstrate the excellent biocompatibility of this D-P_10_C_2_ hydrogel, ascribing to the utilization of biocompatible materials of PVA/CNF and the fully physical crosslinking approach without any toxic small molecules. The bio-adhesion behavior of this hydrogel patch was firstly assessed by evaluating the adhesive performance of L929 fibroblasts on the surface of D-P_10_C_2_ hydrogel, because the fibroblasts were the main component of connective tissue during wound healing [49]. The fibroblasts were firstly seeded on the surface of hydrogel sample and co-cultured for 24 h, subsequently using the Calcein AM/PI reagent to stain the adhesive cells (Fig. 6d–e). Compared to the dense cells on the control sample of cell cultured plate, only few cells were attached on the surface of D-P_10_C_2_ hydrogel. The relative area coverage of adhesive cells for D-P_10_C_2_ hydrogel was just 0.44 % (Fig. 6f). This excellent resistance to fibroblasts adhesion was ascribed to the negative charges of carboxyl groups for CNF which was in the D-P_10_C_2_ hydrogel backbone, which generating electrostatic repulsion with negatively charged cell membranes [14]. This good anti-adhesion of fibroblasts is beneficial to the inhibition of connective tissues between the surgical site and other viscera.

### 3.4 In vivo anti-adhesion efficacy and inflammatory regulation of the hydrogel patch

**Fig. 7:**
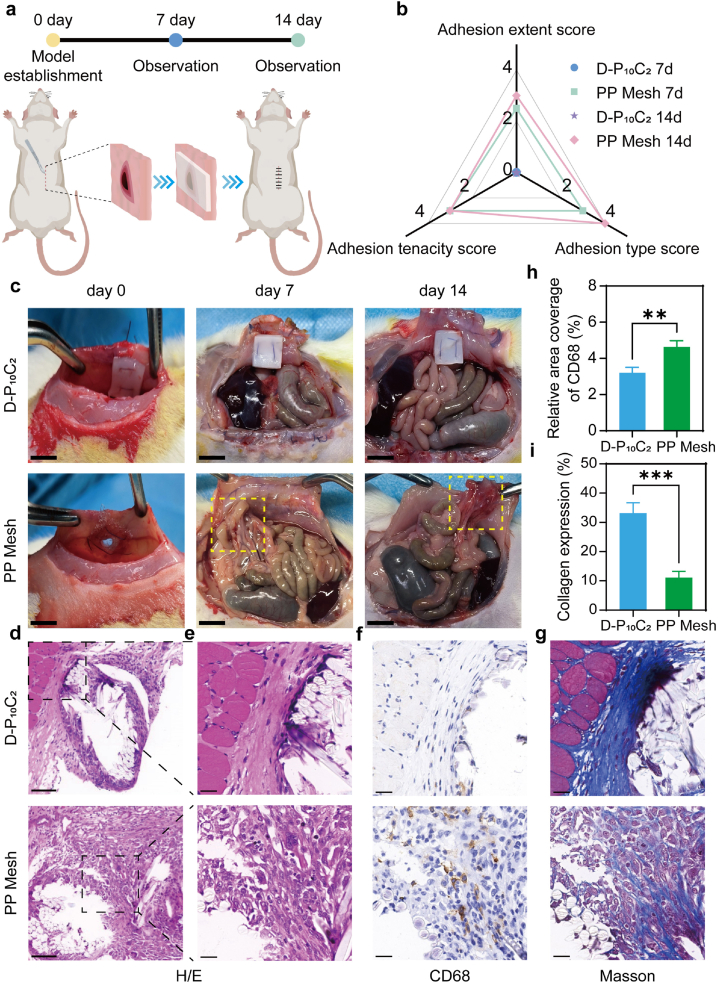
a) Schematic illustration of the establishment and observation of the rat abdominal wall defect model. b) Adhesion scores of wound tissues for different groups on days 7 and 14 after surgery. c) Photos of visceral adhesion formation of abdominal wall defects for different groups on days 7 and 14 after surgery. Scale bar: 1 cm. H/E staining images on day 14 after surgery of wound tissues for different groups. Scale bar: 100 μm in (d) and 25 μm in (e). f) Immunohistochemical staining images of CD68 on day 14 after surgery of wound tissues for different groups. Scale bar: 25 μm. g) MTS images on day 14 after surgery of wound tissues for different groups. Scale bar: 25 μm. h) Quantitative analysis of relative area coverage of CD68 for different groups. i) Quantitative analysis of relative area coverage of collagen deposition for different groups. Data are shown as mean ± SD (*n* = 3). ∗p < 0.05, ∗∗p < 0.01, ∗∗∗p < 0.001.

The anti-adhesion performance of D-P_10_C_2_ hydrogel was systematically evaluated by establishing the rat abdominal defect model. A full-thickness side-wall defect with 8 mm was created by using the circular punch. And the defects were treated by different patches including the D-P_10_C_2_ hydrogel and the commercial PP mesh. The rats were euthanized and observed on the 7th day and 14th day after surgery (Fig. 7a). The adhesion levels were quantified according to a standard adhesion scoring system, including the adhesion tenacity, adhesion type and adhesion extent [31]. On days 7 and 14 postoperative, no any adhesion was found for the D-P_10_C_2_ hydrogel treated defects, and the implanted D-P_10_C_2_ patch retained its original shape and was still fixed on the side-wall because of its excellent mechanical performance and anti-swelling ability in the wet environment. And the adhesion extent score was as low as 0, clearly indicating the great anti-adhesion property of D-P_10_C_2_ hydrogel. In contrast, the PP mesh was completely enclosed by tissues, and severe abdominal and visceral adhesion appeared for the PP mesh treated defects at 7th and 14th day, and the corresponding adhesion extent score were 2.5 and 3, respectively (Fig. 7b–c).

Hematoxylin-eosin (H&E) staining was used to evaluate the inflammatory response of the local surgical site after 14th day post-operative. As shown in Fig. 7d–e, evident infiltration of inflammatory cells was found around the adhesive tissues for the PP mesh treated defect. The mechanical mismatch of PP mesh and the soft abdominal tissue is easy to induce the friction between the mesh and intestinal mucosa (Fig. S4), which leading to the accumulation of inflammatory cells [14]. However, the inflammation around the D-P_10_C_2_ treated wound was alleviated, which revealing its potential to inhibit visceral adhesion with high bio-safety. Additionally, the immunohistochemistry staining figures showed much more CD68 labeled macrophage appeared for PP mesh group compared to that of D-P_10_C_2_ group, and the semi-quantitative test demonstrated the expression of CD68 for D-P_10_C_2_ group was significantly lower than that of PP mesh group (Fig. 7f–h and S4). This result was ascribed to the great bio-compatibility and similar soft property to bio-tissues for D-P_10_C_2_ hydrogel, which could reduce the irritation to the damaged tissues and thereby reducing inflammation. The further Masson's trichrome staining indicated the much more deposition of collagen was observed for D-P_10_C_2_ group, demonstrating its potential pro-healing performance for the soft tissue defects (Fig. 7g–i).

**Fig. 8:**
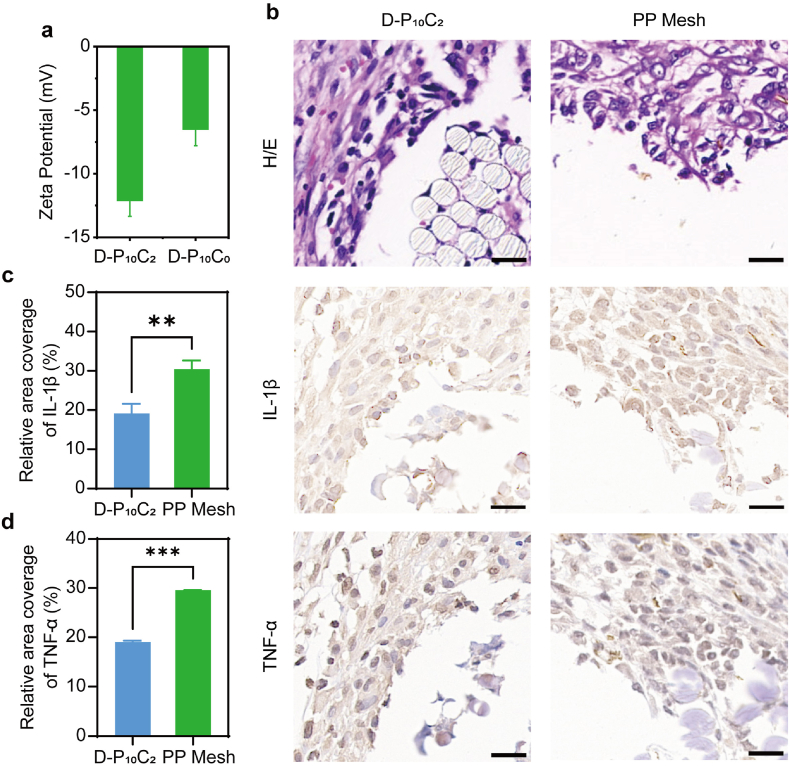
a) Histograms depicting the zeta potentials of D-P_10_C_2_ and D-P_10_C_0_ hydrogels. b) H/E staining, immunohistochemical staining images of IL-1β and TNF-α on day 7 after surgery of wound tissues for different groups. Scale bar: 20 μm. c) Quantitative analysis of relative area coverage of IL-1β for different groups. d) Quantitative analysis of relative area coverage of TNF-α for different groups. Data are shown as mean ± SD (*n* = 3). ∗p < 0.05, ∗∗p < 0.01, ∗∗∗p < 0.001.

The inflammatory response of the surgical site plays the key role for the adhesion formation and tissue pro-healing. Many kinds of proinflammatory cytokines are positively charged. Hence, the negatively charged patches have the potential to effectively capture the positively charged proinflammatory cytokines, which is beneficial for safe healing process. In order to evaluating the inflammatory regulation property of D-P_10_C_2_ hydrogel, its surface charge was firstly detected. Compared to the D-P_10_C_0_ hydrogel, the D-P_10_C_2_ hydrogel exhibits much more negative potential because of the incorporation of CNF which has abundant carboxyl groups (Fig. 8a). In this work, proinflammatory cytokines of IL-1β and TNF-α were chosen as the example to detect the inflammatory regulation ability of D-P_10_C_2_ hydrogel. The immunohistochemical staining images showed the D-P_10_C_2_ hydrogel treated defect significantly reduced the expression of IL-1β and TNF-α compared to that of PP mesh group. And the calculated results of relative area of coverage for these two kinds of cytokines were consistent with this result, validating the high potential for regulating the inflammatory response of this D-P_10_C_2_ hydrogel (Fig. 8b–d).

## 4 Conclusion

In this work, a high-performance anti-adhesion hydrogel patch was created by the facile freeze-casting technique, which exhibited great anti-swelling performance and unique inflammatory regulation. This hydrogel not only possessed comparable mechanical property with soft tissues which could avoid the potential FBRs because of the mechanical mismatch, but also displayed excellent anti-fatigue and anti-tearing performance which could effectively resist the abdominal pressure and surgical suturing tension, ascribing to the highly oriented microstructure and abundant path of energy dissipation within the network. Specifically, the swelling ratio of this hydrogel in PBS was as low as 5.9 %, which was superior to other reported bio-medical hydrogels, while simultaneously keeping the high water-content (89.8 %). This remarkable compatibility of low swelling ratio and high water-content was difficult to achieve for the traditional anti-swelling hydrogels. Meanwhile, this hydrogel owns excellent bio-compatibility with high cell viability and low hemolysis ratio because of the biocompatible PVA/CNF backbone and the completely physical crosslinking strategy. Impressively, this hydrogel displays effective anti-adhesion ability in the rat abdominal defect model. On one hand, the superior mechanical stability in wet environment and the resistance to fibroblasts adhesion made it as a strong physical barrier to isolate the defect and other viscera. On the other hand, the negatively charged hydrogel patch could regulate the inflammatory response by capturing the positively charged proinflammatory cytokines. Our work provides a facile approach to make the effective anti-adhesion hydrogel patch with significant value of clinical translation.

## CRediT authorship contribution statement

**Jianyu Qiu:** Writing – original draft, Validation, Methodology, Formal analysis, Data curation. **Xitong Kang:** Visualization, Validation, Software, Methodology, Investigation. **Tao Liu:** Supervision, Project administration, Investigation, Funding acquisition, Conceptualization. **Jing Liu:** Validation, Methodology. **Huansheng Liu:** Visualization, Software. **Xiyang Zhao:** Software, Methodology, Conceptualization. **Yong Li:** Visualization, Methodology. **Qi Liu:** Software, Methodology. **Zhenzhen Nong:** Visualization, Validation. **Qingwen Wang:** Resources, Funding acquisition. **Zhenzhen Liu:** Writing – review & editing, Visualization, Project administration, Methodology, Formal analysis, Conceptualization.

## Declaration of competing interest

The authors declare that they have no known competing financial interests or personal relationships that could have appeared to influence the work reported in this paper.

## Data Availability

Data will be made available on request.
