# Supplementary material for: A non-swellable, anisotropic hydrogel patch with superior mechanical stability for internal anti-adhesion via physical barrier and inflammation regulation
Source: Mater Today Bio. 2025 Jun 25;33:102017. doi: 10.1016/j.mtbio.2025.102017 (PMC12241401; doi:10.1016/j.mtbio.2025.102017)
Supplement: Multimedia component 5 [file mmc5.docx]

Supporting information

A non-swellable, anisotropic hydrogel patch with superior mechanical stability for internal anti-adhesion via physical barrier and inflammation regulation

Jianyu Qiu ^c 1^, Xitong Kang ^c 1^, Tao Liu ^b^ *, Jing Liu ^c^, Huansheng Liu ^c^, Xiyang Zhao ^c^, Yong Li ^c^, Qi Liu ^b^, Zhenzhen Nong ^b^, Qingwen Wang ^c^, Zhenzhen Liu ^ac^ *

^a^ The Third Affiliated Hospital, School of Biomedical Engineering, Guangzhou Medical University, Guangzhou 511436, China

^b^ College of Food Science, South China Agricultural University, Guangzhou 510642, China

^c^ Institute of Biomass Engineering, College of Materials and Energy, South China Agricultural University, Guangzhou 510642, China

^1^ Equal contributions to this work.


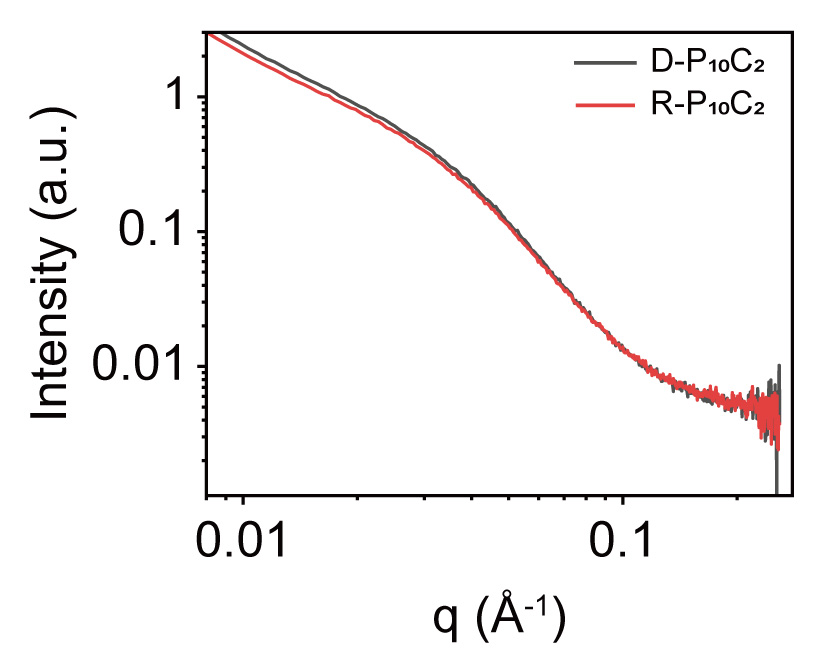


**Figure S1.** The scattering intensity versus scattering vector q during SAXS test of D-P_10_C_2_ and R-P_10_C_2_ hydrogel.


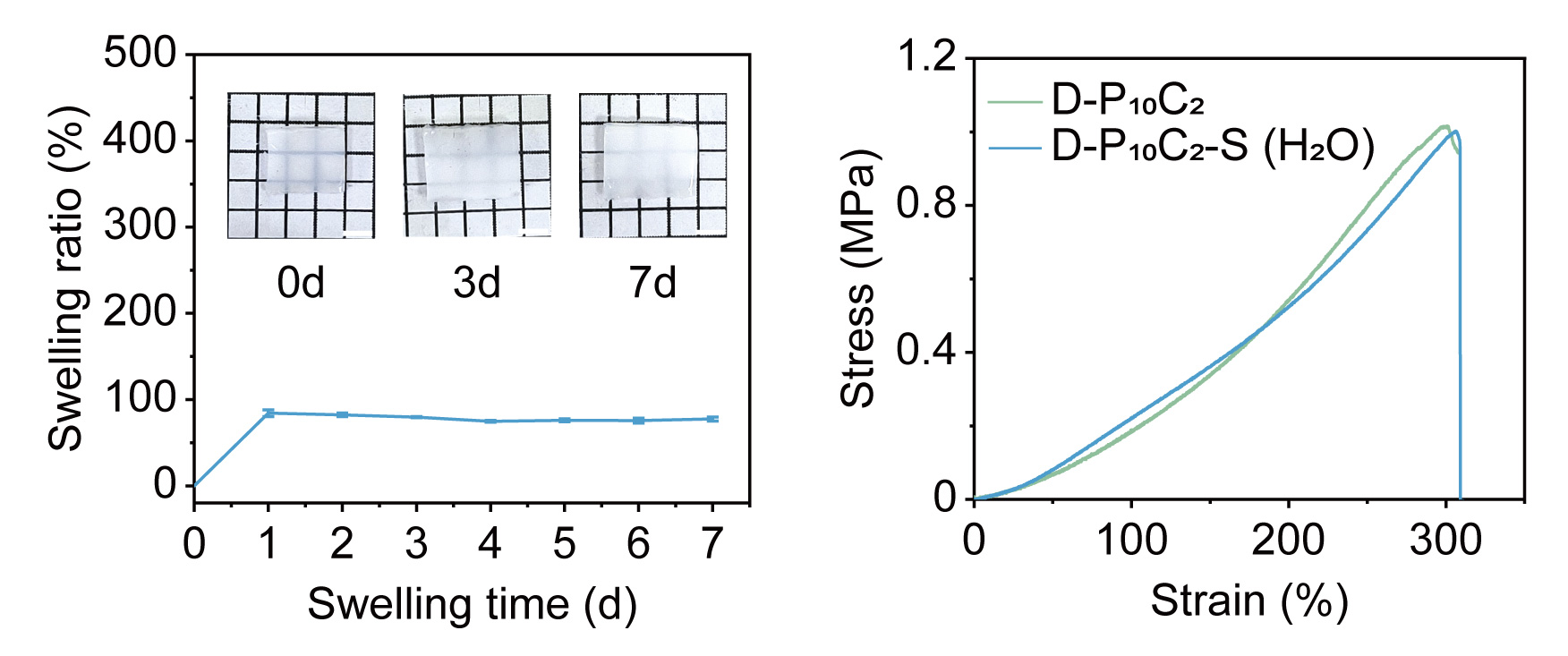


**Figure S2.** Swelling ratio of D-P_10_C_2_ hydrogel immersed in di-H_2_O over 7 days and the corresponding photos of the swelled D-P_10_C_2_ hydrogel at different time intervals. Scale bar: 4 mm.


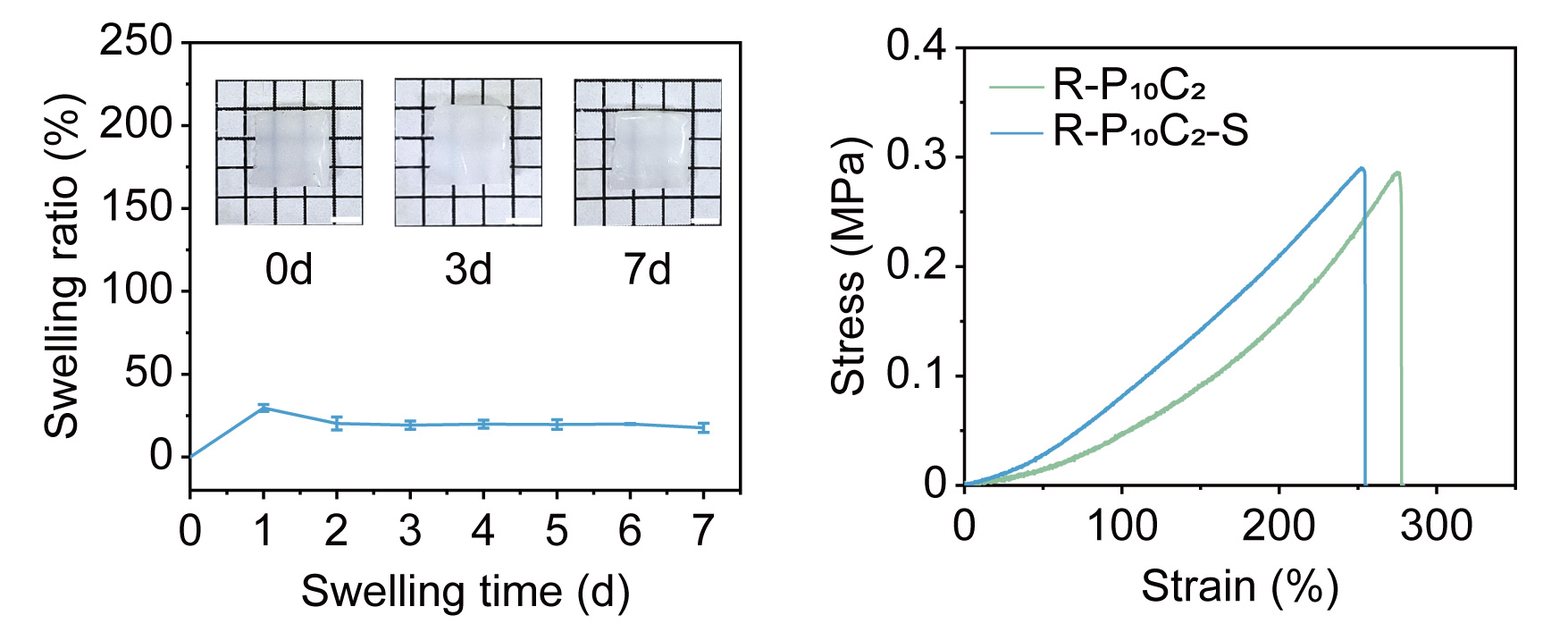
**Figure S3.** Swelling ratio of R-P_10_C_2_ hydrogel immersed in PBS solution over 7 days and the corresponding photos of the swelled R-P_10_C_2_ hydrogel at different time intervals. Scale bar: 4 mm


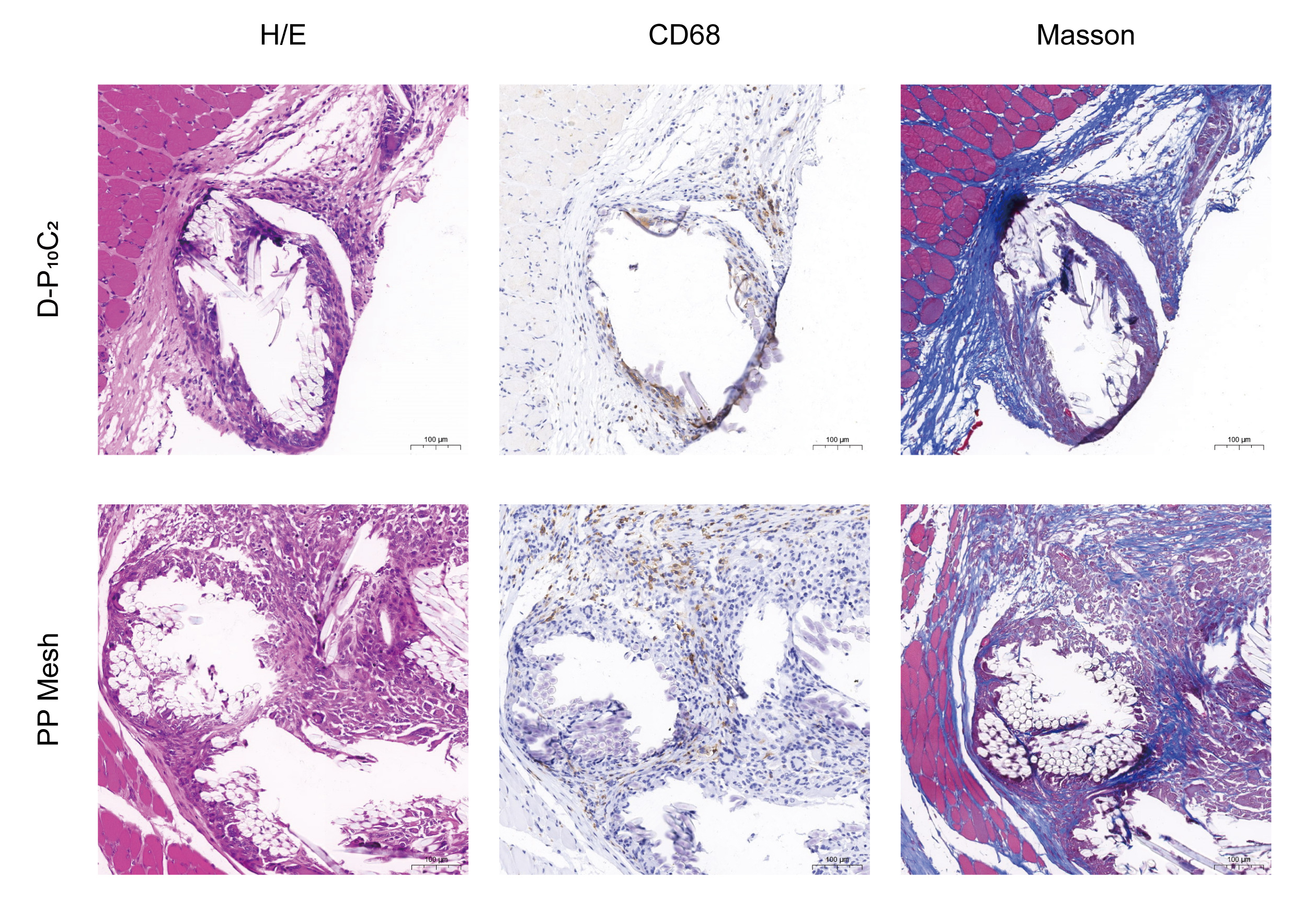


**Figure S4.** The H/E staining, CD68 Immunohistochemical staining, and Masson’s trichrome staining images on day 14 after surgery of wound tissues for D-P_10_C_2_-treated and PP mesh-treated groups, respectively, scale bar: 100 μm.

**Table S1.** The comparison of water content and anti-swelling coefficient for this work with other related hydrogels.

| Ref | Hydrogel | Soaking time | Soaking temperature | Preparation method | Water  content | Anti-swelling  coefficient |
| --- | --- | --- | --- | --- | --- | --- |
| This work | D-P_10_C_2_ | 7 d | 37℃ | freeze-casting | 89.8% | 0.91 |
| 1 | STHB 10L3P | 21d | 37℃ | Room temperature  crosslinking | 87% | 0.63 |
| 2 | Z-1 hydrogel | 80 h | 37℃ | Thiol-ene addition reaction | 85% | 0.13 |
| 3 | PNAAA-25 | 3 d | / | Thermal polymerization | 79.9% | 0.09 |
| 4 | AUE-3 | 14 d | / | Photopolymerization +  solution immersing | 50% | 0.83 |
| 5 | PAA-GT-HBPC_6_ | 3 d | 37℃ | Photopolymerization + SurfaceModification | 58.2% | 0.83 |
| 6 | GAE | 3 d | / | Photopolymerization | 73.9% | 0.62 |
| 7 | Thiolactone Gel | 4 d | 37℃ | Photopolymerization | 59.4% | 0.92 |
| 8 | PNA-G | 12 h | RT | Radical Polymerization | 65.5% | 0.09 |

**Table S2.** The comparison of remaining tensile stress and anti-swelling coefficient for this work with other related hydrogels.

| Ref | Hydrogel | | Soaking time | Soaking temperature | Preparation method | Remaining tensile stress | Anti-swelling  coefficient |
| --- | --- | --- | --- | --- | --- | --- | --- |
| This work | | D-P_10_C_2_ | 7 d | 37℃ | freeze-casting | 99.5% | 0.91 |
| 9 | PAM/XG | | 8 d | RT | Thermal polymerization | 38.8%  48.8%  50% | 0.25  0.23  0.22 |
| 10 | PVA/CS | | 12 h | / | 3D printing + freeze–thawing | 46.2% | 0.36 |
| 11 | Agar/pAAEE DN | | 2 d | RT | heating–cooling + photopolymerization | 13.1%  17.2% | 0.19  0.23 |
| 12 | PVA/CS/Cit | | 15 d | / | freeze–thawing + solution immersing | 41.1% | 0.44 |
| 13 | CS/MMT | | 12 d | 25℃ | Radical Polymerization | 9.8%  5.5% | 0.61  0.76 |
| 14 | CS/Sul | | 7 d | 37℃ | photopolymerization | 70% | 0.55 |
| 15 | k-G/pAAm | | 1 d | 30℃  40℃ | Photopolymerization + solution immersing | (18.5%, 30℃)  (25.6%, 40℃) | 0.42  0.42 |
| 16 | G3P7 | | 7 d | 37℃ | Physical crosslinking + solvent exchange | 75.3% | 0.95 |

**References**

[1] G.U. Ruiz-Esparza, X. Wang, X. Zhang, S. Jimenez-Vazquez, L. Diaz-Gomez, A.-M. Lavoie, S. Afewerki, A.A. Fuentes-Baldemar, R. Parra-Saldivar, N. Jiang, N. Annabi, B. Saleh, A.K. Yetisen, A. Sheikhi, T.H. Jozefiak, S.R. Shin, N. Dong, A. Khademhosseini, Nanoengineered Shear-Thinning Hydrogel Barrier for Preventing Postoperative Abdominal Adhesions, Nano-Micro Lett. 13(1) (2021) 212. <https://doi.org/10.1007/s40820-021-00712-5>.

[2] Q. Guo, H. Sun, X. Wu, Z. Yan, C. Tang, Z. Qin, M. Yao, P. Che, F. Yao, J. Li, In Situ Clickable Purely Zwitterionic Hydrogel for Peritoneal Adhesion Prevention, Chem. Mater. 32(15) (2020) 6347-6357. <https://doi.org/10.1021/acs.chemmater.0c00889>.

[3] J. Yu, K. Wang, C. Fan, X. Zhao, J. Gao, W. Jing, X. Zhang, J. Li, Y. Li, J. Yang, W. Liu, An Ultrasoft Self-Fused Supramolecular Polymer Hydrogel for Completely Preventing Postoperative Tissue Adhesion, Adv. Mater. 33(16) (2021) 2008395. <https://doi.org/https://doi.org/10.1002/adma.202008395>.

[4] J. Yang, W. Liu, W. Wang, A supramolecular hydrogel leveraging hierarchical multi-strength hydrogen-bonds hinged strategy achieving a striking adhesive-mechanical balance, Bioact. Mater. 43 (2025) 32-47. <https://doi.org/https://doi.org/10.1016/j.bioactmat.2024.09.014>.

[5] Y. Liang, H. Xu, Q. Han, M. Xu, J. Zhang, J. Wang, X. Liu, Z. Yin, B. Guo, A Janus hydrogel sealant with instant wet adhesion and anti-swelling behavior for gastric perforation repair, Nano Today 54 (2024) 102105. <https://doi.org/https://doi.org/10.1016/j.nantod.2023.102105>.

[6] H. An, M. Zhang, Z. Huang, Y. Xu, S. Ji, Z. Gu, P. Zhang, Y. Wen, Hydrophobic Cross-Linked Chains Regulate High Wet Tissue Adhesion Hydrogel with Toughness, Anti-hydration for Dynamic Tissue Repair, Adv. Mater. 36(8) (2024) 2310164. <https://doi.org/https://doi.org/10.1002/adma.202310164>.

[7] J. Han, J. Park, R. Bhatta, Y. Liu, Y. Bo, J. Zhou, H. Wang, A double crosslinking adhesion mechanism for developing tough hydrogel adhesives, Acta Biomater. 150 (2022) 199-210. <https://doi.org/https://doi.org/10.1016/j.actbio.2022.07.028>.

[8] L. Feng, W. Shi, Q. Chen, H. Cheng, J. Bao, C. Jiang, W. Zhao, C. Zhao, Smart Asymmetric Hydrogel with Integrated Multi-Functions of NIR-Triggered Tunable Adhesion, Self-Deformation, and Bacterial Eradication, Adv. Healthc. Mater. 10(19) (2021) 2100784. <https://doi.org/https://doi.org/10.1002/adhm.202100784>.

[9] N. Yuan, L. Xu, H. Wang, Y. Fu, Z. Zhang, L. Liu, C. Wang, J. Zhao, J. Rong, Dual Physically Cross-Linked Double Network Hydrogels with High Mechanical Strength, Fatigue Resistance, Notch-Insensitivity, and Self-Healing Properties, ACS Appl. Mater. Interfaces 8(49) (2016) 34034-34044. <https://doi.org/10.1021/acsami.6b12243>.

[10] P. Jiang, P. Lin, C. Yang, H. Qin, X. Wang, F. Zhou, 3D Printing of Dual-Physical Cross-linking Hydrogel with Ultrahigh Strength and Toughness, Chem. Mater. 32(23) (2020) 9983-9995. <https://doi.org/10.1021/acs.chemmater.0c02941>.

[11] Y. Zhang, B. Ren, S. Xie, Y. Cai, T. Wang, Z. Feng, J. Tang, Q. Chen, J. Xu, L. Xu, J. Zheng, Multiple Physical Cross-Linker Strategy To Achieve Mechanically Tough and Reversible Properties of Double-Network Hydrogels in Bulk and on Surfaces, ACS Appl. Polym. Mater. 1(4) (2019) 701-713. <https://doi.org/10.1021/acsapm.8b00232>.

[12] C. Luo, Y. Zhao, X. Sun, B. Hu, Developing high strength, antiseptic and swelling-resistant polyvinyl alcohol/chitosan hydrogels for tissue engineering material, Mater. Lett. 280 (2020) 128499. <https://doi.org/https://doi.org/10.1016/j.matlet.2020.128499>.

[13] X. Su, S. Mahalingam, M. Edirisinghe, B. Chen, Highly Stretchable and Highly Resilient Polymer–Clay Nanocomposite Hydrogels with Low Hysteresis, ACS Appl. Mater. Interfaces 9(27) (2017) 22223-22234. <https://doi.org/10.1021/acsami.7b05261>.

[14] Y. Yang, X. Wang, F. Yang, L. Wang, D. Wu, Highly Elastic and Ultratough Hybrid Ionic–Covalent Hydrogels with Tunable Structures and Mechanics, Adv. Mater. 30(18) (2018) 1707071. <https://doi.org/https://doi.org/10.1002/adma.201707071>.

[15] H.C. Yu, C.Y. Li, M. Du, Y. Song, Z.L. Wu, Q. Zheng, Improved Toughness and Stability of κ-Carrageenan/Polyacrylamide Double-Network Hydrogels by Dual Cross-Linking of the First Network, Macromolecules 52(2) (2019) 629-638. <https://doi.org/10.1021/acs.macromol.8b02269>.

[16] X. Liu, X. Qiu, L. Nie, B. Zhou, P. Bu, Y. Li, X. Xue, B. Tang, Q. Feng, K. Cai, Nonswellable Hydrogel Patch with Tissue-Mimetic Mechanical Characteristics Remodeling In Vivo Microenvironment for Effective Adhesion Prevention, ACS Nano 18(27) (2024) 17651-17671. <https://doi.org/10.1021/acsnano.4c02321>.
